# Epidemiological and molecular updates on hookworm species in dogs from southern Italy

**DOI:** 10.1186/s12917-023-03765-3

**Published:** 2023-10-13

**Authors:** Sergio Illiano, Lavinia Ciuca, Maria Paola Maurelli, Paola Pepe, Valeria Caruso, Antonio Bosco, Saverio Pennacchio, Ruggero Amato, Marina Pompameo, Laura Rinaldi

**Affiliations:** 1 Department of Veterinary Medicine and Animal Production, University of Naples Federico II, Via Delpino, 1, 80137 Naples, Italy; 2 ASL Naples 1 Centro, Veterinary Hospital, Via Marco Rocco Di Torrepadula, 13, 80145 Naples, Italy

**Keywords:** Dogs, Hookworms, *Uncinaria stenocephala*, *Ancylostoma caninum*, Southern Italy, Molecular identification, Ten-year retrospective analysis

## Abstract

**Background:**

The zoonotic hookworms *Ancylostoma caninum* and *Uncinaria stenocephala* are widespread soil-transmitted helminths in dogs in Europe. Given the veterinary and public health importance of hookworms in dogs and the recent changes in the molecular epidemiology of some species, there is a need to continuously monitor the epidemiological and molecular prevalence of these parasites also at the “local” level. The present study aimed to update the epidemiological scenario of hookworm infections in both owned and stray dogs in southern Italy and to discriminate between different hookworm species (*A. caninum* and *U. stenocephala)* through molecular analyses. For this purpose, a retrospective analysis was performed over 10 years (2011–2021), including a total of 7008 owned dogs and 5642 stray dogs referred to our laboratory for copromicroscopic examinations. Moreover, 72 faecal samples, from dogs naturally infected by hookworms, were used to discriminate between *A. caninum* and *U. stenocephala* using two PCR protocols. Prior to molecular analyses, a subsample of 40/72 positive faecal samples was used for morphometric investigations on hookworm eggs.

**Results:**

The results of the ten-year retrospective analysis (2011–2021) showed an overall prevalence of hookworm infection of 9.16%, specifically 5.1% in owned dogs and 14.2% in stray dogs. Logistic regression showed a significant association between positivity to hookworms and the variable “puppies” both in stray (13.84%; OR = 2.4) and owned (7.07%; OR = 2.2) dogs. The results of molecular analyses showed that positivity was confirmed only in 21/72 samples, specifically, 6 samples using protocol A and 19 with protocol B. Sequencing revealed 15 samples positive to *U. stenocephala* and 6 to *A. caninum*.

**Conclusions:**

The findings of this study showed a high prevalence of hookworm infections in dogs in southern Italy, updating the epidemiological scenario of the last decade. Moreover, the results of the study revealed the first identification of hookworm species in dogs in Italy by molecular studies, highlighting that *U. stenocephala* is more prevalent than *A. caninum.*

## Background

Among the intestinal parasites that infect dogs, the hookworms *Ancylostoma caninum* and *Uncinaria stenocephala* play an important role in the health and welfare of canine populations worldwide, as well as in public health, due to their zoonotic potential [1–3]. Both pathogens might cause *larva migrans* syndrome or “ground itch” in humans [4, 5]. Moreover, the possibility of causing eosinophilic enteritis in human hosts which determines diarrhea, abdominal pain and weight loss has also been described [6].

The main source of infection in dogs is the soil contaminated with eggs excreted in dog faeces, where larvae hatch and develop to the infective stage L3 at suitable temperatures and humidity rates. Infection occurs mainly by percutaneous penetration of L3 or their ingestion *per os* [7]. In addition, hookworms are known to cause anaemia and hypoproteinemia in dogs, especially in puppies [8, 9].

Hookworms are common parasites in dogs and wild carnivores throughout the world, with prevalence values varying by climatic regions and dog population. In Europe, prevalence rates of hookworm infections in dogs range from 1.2% to 34% [9–13]. In particular, in Italy, hookworm infections have been reported in many studies, with high prevalence rates in stray (67.7%) and owned dogs (18.9%) in the southern area [14, 15], followed by prevalence rates between 0–9.3% in stray dogs and 0.4–3.6% in owned dogs in the northern area [16–19]. However, there are few studies to support the identification of hookworm species in dogs around the world. For example, in Central Europe, according to a recent study, *U. stenocephala* infection seems to be more prevalent than *A. caninum* infection in dogs [20]. On the other hand, in Africa [21–23], Asia [24–26] and Brasil [27], the occurrence of *A. caninum* was reported with higher frequency than *U. stenocephala* species. Moreover, the data also revealed mixed infections with other hookworm species such as *Ancylostoma ceylanicum* or *Ancylostoma braziliense*. Hence, considering the above information and the impact of hookworm infections on veterinary and public health, it should be imperative to continuously monitor the prevalence of hookworms in dogs in Europe and Italy. In addition, the fact that there are few studies [20, 28] reporting the differentiation between hookworm species in dogs in Europe, but no study conducted so far in Italy, demands future research to estimate and evaluate the zoonotic aspects of hookworm infections. Therefore, the present study aims to update the epidemiological scenario of hookworm infections in owned and stray dogs in southern Italy by performing a retrospective analysis of prevalence over ten years (2011–2021) and a molecular study to identify the occurence of *A. caninum* and *U. stenocephala*.

## Results

The results of the ten-year retrospective analysis (2011–2021) in southern Italy showed an overall prevalence of hookworm infections of 9.16% (1159/12650; 95% CI = 8.67–9.68) in owned and stray dogs. More specifically, a prevalence of 5.1% (355/7008; 95% CI = 4.57–5.61) was found in owned dogs with a mean egg shedding of 222.9 eggs per gram (EPG) of feaces (2–6,440 EPG; SD = 601.07) and 14.2% (804/5642; 95% CI = 13.35–15.20) in stray dogs with a mean EPG of 20.5 (2–556 EPG; SD = 39.92). Prevalence values per year (2011–2021) of hookworm infection, both in owned and stray dogs are reported in Table 1. Co-infections with other helminths and protozoa (*Trichuris, Toxocara, Capillaria, Isospora, Giardia*) were also found (data not showed). Almost all dogs were apparently healthy (80%), while in a few cases abnormalities in faecal consistency such as diarrhea or the presence of blood (20%) were observed.

**Table 1:** Results of prevalence (%) and 95% confidence of interval (95% CI) for hookworm infections in owned and stray dogs included in the study-period from 2011 until 2021

Year of testing	Owned dogs		Stray dogs		Overall	
	**Positive /total samples**	**Prevalence % (95% CI)**	**Positive /total samples**	**Prevalence % (95% CI)**	**Positive /total samples**	**Prevalence % (95% CI)**
**2011**	13/171	7.60 (4.28–12.92)	104/848	12.26 (10.17–14.71)	117/1019	11.48 (9.62–13.64)
**2012**	9/270	3.33 (1.64–6.45)	88/624	14.10 (11.52–17.14)	97/894	10.85 (8.93–13.12)
**2013**	15/378	3.97 (2.32–6.60)	78/604	12.91 (10.40–15.91)	93/982	9.47 (7.75–11.52)
**2014**	13/472	2.75 (1.54–4.79)	96/630	15.24 (12.57–18.34)	109/1102	9.89 (8.22–11.84)
**2015**	22/707	3.11 (2.01–4.75)	40/266	15.04 (11.08- 20.04)	62/973	6.37 (4.96–8.14)
**2016**	41/583	7.01 (5.15–9.50)	38/214	17.76 (13.02–23.69)	79/797	9.91 (7.97–12.25)
**2017**	130/1001	12.99 (11–15.26)	42/220	19.09 (14.25–25.04)	172/1221	14.09 (12.21–16.19)
**2018**	10/930	1.08 (0.55–2.04)	54/332	16.27 (12.55–20.78)	64/1262	5.07 (3.96–6.47)
**2019**	21/815	2.58 (1.64–3.98)	21/184	11.41 (7.37–17-13)	42/999	4.20 (3.08–5.69)
**2020**	36/522	6.90 (4.94–9.51)	118/817	14.44 (12.14–17.09)	154/1339	11.50 (9.87–13.36)
**2021**	45/1159	3.88 (2.88–5.20)	125/903	13.84 (11.69–16.31)	170/2062	8.24 (7.11–9.54)
**Total**	355/7008	5.1 (4.57–5.61)	804/5642	14.2 (13.35–15.20)	1159/12650	9.16 (8.67–9.68)

The results of the Chi-square test for the different variables considered (gender, age and dog breed size) are reported in Tables 2 and 3. Logistic regression revealed a significant association between positivity to hookworms and the variable “puppies” in both stray (13.84%; OR = 2.4; 95%CI = 12.50–15.21; *P* = 0.004) and owned (7.07%; OR = 2.2; 95%CI = 6.12–8.14 *P* = 0.000) dogs. Regarding the excretion of hookworm eggs in owned dogs, 193/355 (54.4%) were allocated in group A (2–50 EPG), 53/355 (14.9%) in group B (52–100 EPG), 69/355 (19.4%) in group C (102–500 EPG), 21/355 (5.9%) in group D (502–1000 EPG) and 19/355 (5.4%) in group E (≥ 1002 EPG); while, in stray dogs 759/804 (94.4%) were allocated in group A (*P* < 0.005), 32/804 (3.9%) in group B, 11/804 (1.4%) in group C, 2/804 (0.3%) in group D and 0/804 in group E.

**Table 2:** Results of the positivity to hookworms (prevalence %, 95% confidence interval (95% CI), *p*-value) for the variables gender, age, breed size in all stray dogs included in the study (period 2011–2021)

**Total stray dogs analysed = 5642**		**Hookworms**	
	**No. analysed**	**No. positive**	**Prevalence %, 95% CI, *p*-value**
**Gender**			
**Male**	2583	345	**13.36 (12.08–14.74)**
**Female**	3059	459	**15 (13.77–16.33)**
			***P* = *0.078***
**Age**			
** Puppies (< 12 months)**	2428	336	**13.84 (12.50–15.29)**
** Young (1–3 years)**	1766	258	**14.61 (13.01–16-36)**
** Adult (4–6 years)**	745	124	**16.64 (14.08–19.56)**
** Old (7–10 years)**	577	72	**12.48 (9.95–15.52)**
** Very old (> 10 years)**	126	14	**11.11 (6.43–18.25)**
			***P* = *0.004***
**Breed size**			
**Small**	1457	206	**14.14 (12.41–16.06)**
**Medium**	3245	464	**13.55 (12.43–14.75)**
**Large**	940	134	**14.26 (12.12–16.69)**
			***P* = *0.989***

**Table 3:** Results of the positivity to hookworms (prevalence %, 95% confidence interval (95% CI), p-value) for the variables gender, age, breed size in all owned dogs included in the study (2011–2021)

**Total owned dogs analysed = 7008**		**Hookworms**	
	**No. analysed**	**No. positive**	**Prevalence %, 95% CI, *p*-value**
**Gender**			
**Male**	2978	145	**4.78 (4.14–5.72)**
**Female**	4030	210	**5.21 (4.55–5.95)**
			***P* = *0.519***
**Age**			
** Puppies (< 12 months)**	2576	182	**7.07 (6.12–8.14)**
** Young (1–3 years)**	1618	80	**4.94 (3.96–6.15)**
** Adult (4–6 years)**	2106	70	**3.32 (2.62–4.20)**
** Old (7–10 years)**	544	17	**3.13 (1.89–5.06)**
** Very old (> 10 years)**	164	6	**3.66 (1.50–8.15)**
			***P* = *0.000***
**Breed size**			
**Small**	1440	84	**5.83 (4.70–7.20)**
**Medium**	5071	253	**4.99 (4.41–5.63)**
**Large**	497	18	**3.62 (2.22- 5.7)**
			***P* = *0.137***

The results of molecular analyses showed that 21/72 samples were confirmed with both protocols (A, B). Specifically, 6 samples were confirmed with protocol A and 19 with protocol B. In addition, only four samples were resulted positive at both PCR protocols (A, B) Sequencing revealed that 15 samples were identified as *U. stenocephala* (100% identity; MT345056) and 6 samples as *A. caninum* (100% identity; MT1309331). Co-infections with the two hookworm species were not detected in any sample.

**Fig. 1:**
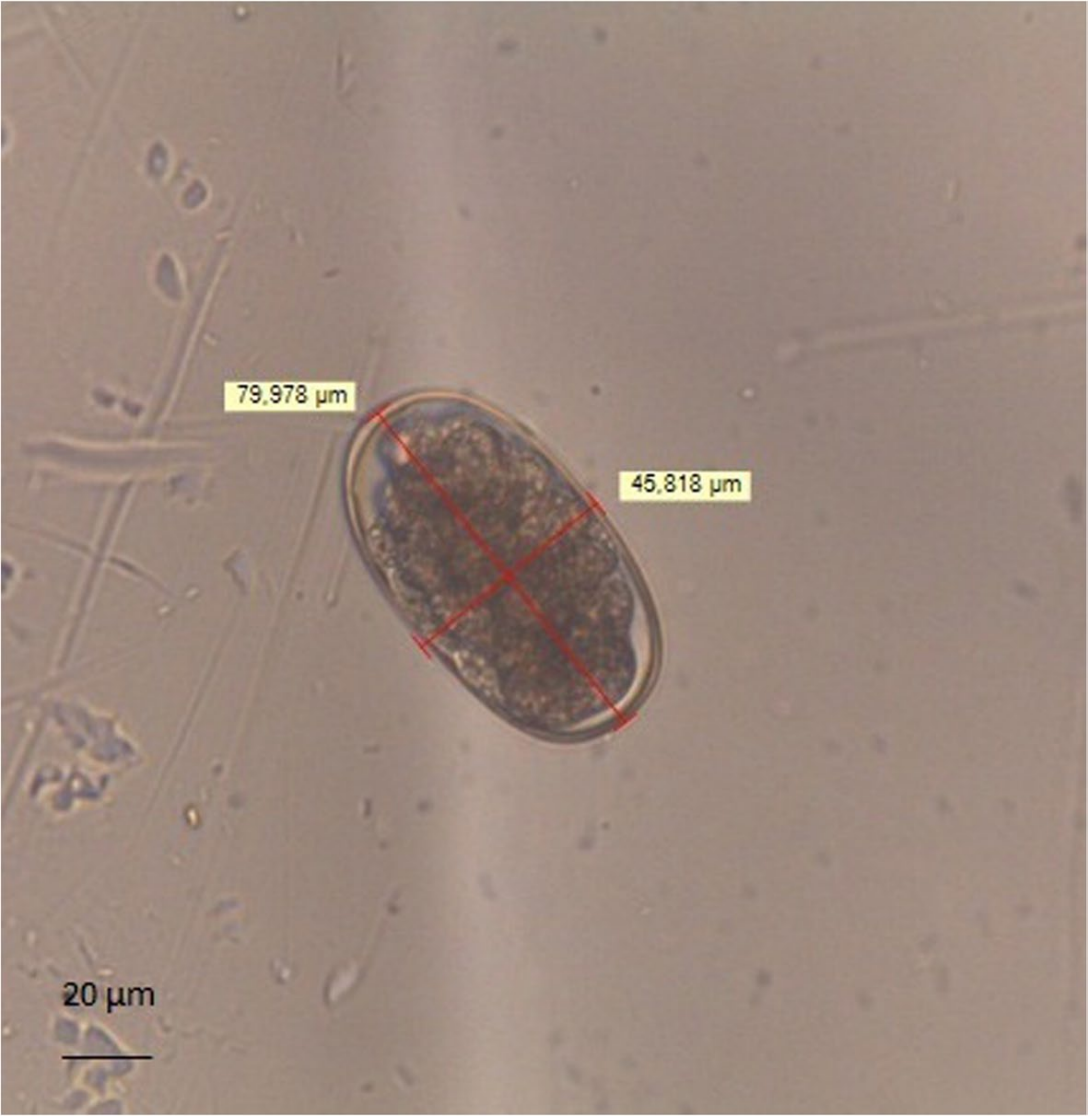
Egg of *Uncinaria stenocephala* (79 µm X 45 µm) with FLOTAC technique

**Fig. 2:**
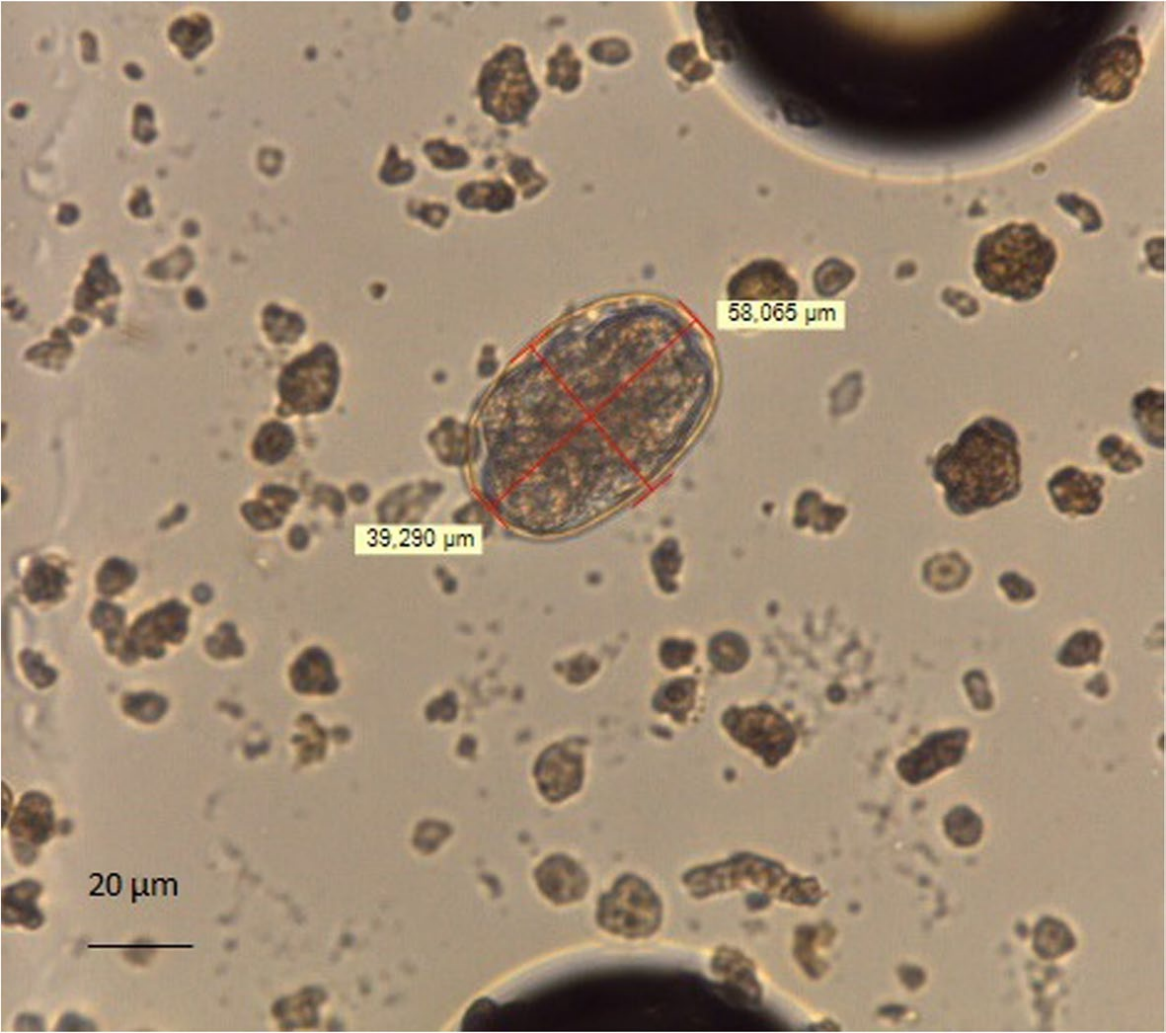
Egg of *Ancylostoma caninum* (58 µm X 39 µm) with FLOTAC technique

The results of morphometric analyses showed that 28/40 hookworm positive samples were similar to *U. stenocephala* (Fig. 1) (major axis of egg = 80.532 ± 3.120 μm; minor axis = 46.591 ± 3.691 μm; perimeter = 214.477 ± 3.703 μm) and 12/40 samples were similar to *A. caninum* (Fig. 2) (major axis of eggs = 66.305 ± 5.675 μm; minor axis = 41.348 ± 4.033 μm; perimeter = 175.375 ± 6.029 μm) [29].

Of the total 40 faecal samples subjected to the morphometric analyses, the results of molecular analysis could be assigned to only 10 samples as follows: 2 samples with protocol A and 8 with protocol B. Finally, the molecular results of the samples confirmed by PCR and sequencing (*N* = 10) agreed with the results of the morphometric analyses, i.e., *U. stenocephala* (*n* = 9) and *A. caninum* (*n* = 1)

## Discussion

Hookworms in dogs cause clinically relevant parasitic infections that are common worldwide, with prevalence rates varying by geographic area and dog population [9–11]. In Asia, Africa, North America and Australia hookworm infection are widespread with different prevalence rates, e.g. 23–79% [30–32], 30–32% [21, 22, 33], 10–91% [34] and 6–10% [35, 36], respectively. The presence of hosts other than dogs, such as foxes and wolves, and climatic conditions favorable for larval development are important factors that could influence the distribution of hookworms in dog populations worldwide [37]. According to some studies conducted in Europe, the prevalence of hookworms ranges from 10 to 12% in foxes [11, 38] and from 30 to 90% in wolves [39, 40], whereas a recent study on intestinal parasites in dogs in cities across Western Europe revealed a hookworm prevalence of 3.2% [13].

In Italy, data on the prevalence of hookworm infections in dogs vary widely from north to south and depend on the diagnostic method used, the study area (rural, urban, and suburban), the dogs’ lifestyle, and the chemoprophylaxis regimes [14–19, 41]. The data obtained in the present study on the overall prevalence of hookworms (9.2%) in dogs in southern Italy is in line with the prevalence (11.6%) obtained in an harmonized survey recently conducted in Italy [15]. As expected, the prevalence was higher in stray dogs (14.2%) than in owned dogs (5.1%), confirming the data of previous studies from the same area [14, 15]. These findings showed that hookworms are still prevalent in dog populations in southern Italy, despite the increased awareness of veterinarians and owners promoted by the national and European guidelines of the European Scientific Counsel Companion Animal Parasites (ESCCAP) [42].

On the other hand, the present study showed greater egg shedding in owned dogs than in stray dogs. According to the authors' knowledge there is no explanation for this result but the only hypothesis that could justify this would be the immunological response induced by the infected stray dogs due to their constant exposure to hookworm eggs, resulting in elimination of low EPG levels than in owned dogs which their exposure to hookworm eggs could be less frequent but with intense elimination of parasitic eggs. Thus, further studies are needed in order to investigate the immunological response both in experimental and naturally infected dogs with hookworms. However one of the limit of this study was the impossibility to highlight the seasonality regarding the shedding of the eggs, as the number of analyzed samples varied greatly in different months and years.

Statistical results of the present study showed that positivity for hookworms was significantly related to the age of infected dogs with higher prevalence in puppies. According to Gates et al. (2009) [43], puppies are more likely to be infected through transmammary route during the lactation period in case of *A. caninum*. However, the possibility of transplacental transmission has not yet been described. In addition, the higher pathogenicity of hookworm species in dogs depending on the age of the dog must be considered. [8]. In fact, puppies affected by hookworms infection usually suffer from diarrhea and anemia and sometimes die in massive infections [8].

The results of the present study showed variable values ​​of hookworm prevalence per year (2011–2021), ranging from 1 to 13% in owned dogs and from 11 to 20% in stray dogs, but no temporal trend was observed. In contrast, retrospective studies conducted in the USA (in 2012–2018 and 2013–2017) [44, 45] and in central Italy (in 2015–2020) [41] showed an increasing trend in the prevalence of hookworm infections (2.02–2.96%, 1.17–2.77%, and 6.8–16.5%) over their study periods. This could be due to multiple factors such as: climatic conditions influencing parasite development in the environment, possible resistance to commercially available anthelmintics [46], the use of copromicroscopic techniques with different detection limits, and the different size of dog population used, all of which must be taken into account.

Although several studies on the prevalence of hookworms in dogs have been carried out in Italy, there are no data on the identification and discrimination of the different hookworm species based on molecular studies. In fact, PCR and sequencing are the only tools available so far for hookworm species identification [20, 26]. This is the first molecular identification of hookworm species in dogs in Italy showing that *U. stenocephala* is more prevalent than *A. caninum* in dogs in southern Italy. It is interesting to note that *U. stenocephala* occurs in regions where the climate is not optimal for its development [20], such as the Mediterranean region. It is likely that our findings are due to both climatic changes and increasing animal movements as a result of globalization. It should also be noted that in the present study, not all the faecal samples which were positive to the hookworm eggs with the FLOTAC technique (*N* = 72), were also positive to the PCR protocols (A and B) (*N* = 21/72) used [47]. Moreover, the DNA used for the PCR analyses was extracted from the faecal samples naturally infected with Ancylostomidae eggs and the positive control was extracted from the *Uncinaria stenocephala* adult. However, there are other molecular studies that showed a high prevalence rates of hookworm infection [21, 22, 24, 27], using the same PCR protocol as described in the present study [21, 22, 24, 27]. This may suggest that either the PCR protocols used in the present study are less sensitive, or that a different substrate for DNA extraction should be considered, e.g. L3 larvae instead of eggs in the faecal samples, as shown in another study [22, 25]. In addition, the low prevalence rates of hookworm infections obtained with PCR analyses, reported herein, are similar with what reported in another study in which was performed a different PCR protocol, but using faecal samples naturally infected with high EPG of hookworms [26, 48]. The reduced detection limit of both PCR protocols (A, B) perfomed in the present study could be explained by two hypotheses: the type of matrix used for the DNA extraction, but perhaps, also the load of eggs excreted by the dogs naturally infected by hookworms. Therefore, further studies are needed to improve the sensitivity of the PCR protocol for hookworm detection, by investigating the detection limit and the best type of sample to use (i.e. faeces with eggs, floated suspension with eggs, L3 larvae). However, both hypotheses above mentioned (using spiked and naturally infected samples with hookworms) will be tested in another upcoming study by the authors of the present study.

The morphometric results obtained in this study agree with previous studies [29] and were also confirmed by the molecular analyses [20]. However, using only morphometric analyses of hookworm eggs, it is not possible to discriminate between different hookworm species. One of the limits of this study is that only a few number of samples were used for the morphometric analysis; in addition, all measurements were performed on samples naturally infected by hookworms without using a positive control from experimental infection. However, differentiation of different hookworm species such as *A. caninum* and *U. stenocephala* could also be possible by identification of L3 [49, 50], which was not performed in the present study.

## Conclusions

In conclusion, the findings of this study revealed a high prevalence of hookworm infections in dogs in southern Italy, and updated the epidemiological scenario of the last decade. This study was the first to identify hookworm species (*A. caninum* and *U. stenocephala*) in dogs in Italy through molecular studies. Further studies are needed, especially to differentiate hookworm species and to develop increasingly sensitive, accurate and point-of-care diagnostics to provide more effective surveillance tools for the protection of human and animal health.

## Methods

### Study design

**Fig. 3:**
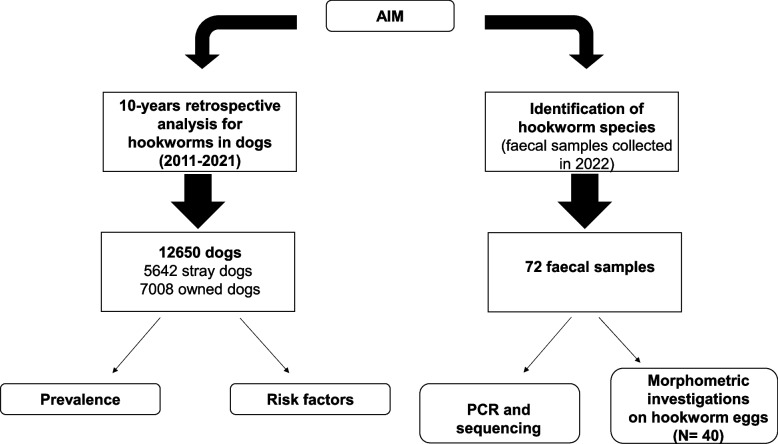
Study design

The study design is summarized in Fig. 3. To update the epidemiological scenario of hookworm infections in dogs in southern Italy, two objectives were pursued. The first objective was to determine the prevalence and analyze the risk factors for hookworm infections in dogs in a Mediterranean area. For this purpose, a retrospective study was conducted analysing ten-years (January 2011-December 2021) of parasitological data from routine diagnostics in dogs from the Campania region (southern Italy). A total of 7,008 owned dogs (males = 4,030; females = 2,978) and 5,642 stray dogs (males = 3,059; females = 2,583) were referred to our laboratories (Parasitology Service of the Veterinary Teaching Hospital, University of Naples Federico II, Italy) for copromicroscopic examination. All faecal samples were analyzed using the FLOTAC technique [51, 52]. Moreover, data on dog’s age, gender, lifestyle (stray/owned dogs) and breed size were collected.

The second objective was to identify hookworm species in dogs in the study area, by morphometric analysis of eggs and confirmation by molecular tests. To this end, all faecal samples collected in 2022 (total number = 1548) that tested positive for hookworms using the FLOTAC technique (*N* = 72) were tested by molecular analyses, using two different protocols: i) protocol A as described by Traub et al., (2004) [47], and; ii) protocol B with some modifications of the previous protocol. In addition, a subsample of 40 of the 72 positive faecal samples was used for morphometric studies of hookworm eggs prior to molecular analyses.

### Laboratory analysis

#### Coprological analyses

Each canine faecal sample (pools of three consecutive days for each faecal sample/per animal) was tested for intestinal parasites (helminths and protozoa) using the FLOTAC dual technique [51, 52] with sodium chloride (specific gravity, s.g. = 1.20) and zinc sulphate (s.g. = 1.20) as flotation solutions. The detection limit (analytic sensitivity) was 2 eggs/oocysts/cysts/larvae per gram (EPG/OPG/CPG/LPG) of faeces.

### Morphometric analysis of hookworm eggs, DNA extraction and molecular analysis

All samples used for molecular analyses (*N* = 72) were previously stored at -20 °C. Therefore, morphometric analyses (major axis, minor axis and perimeter) of 20 hookworm eggs were performed for each of the 40 faecal samples using LAS X Leica software (version 5.0.2, 2021). In addition, the faecal samples that were analysed morphometrically, as well as the samples (*N* = 32) for which the morphometric tests could not be performed (low quantity of faecal samples) were subjected to molecular analyses. Therefore, 72 faecal samples were subjected to DNA extraction using the Fast DNA Stool Kit (Qiagen, Germany) according to the manufacturer's instructions. Specific primers were used for amplification of the ITS1, 5.8S and ITS-2 regions according to Traub et al. (2004) [47] as follows: forward primer RTGHF1 (5′-CGTGCTAGTCTTCAGGACTTTG-3′) and reverse primer RTGHR1 (5′-CGTTGTCATACTAGCCACTGC-3″) for the detection of *Ancylostoma* spp (680 bp region of *A. caninum*). To also confirm the amplification of *Uncinaria stenocepahala*, an adult specimen of *U. stenocepahala* was used in the same PCR protocol. The results showed that the purified PCR product of the adult specimen was confirmed as *U. stenocephala* after sequencing the 680 bp region using the same primers as described above.

Finally, the first PCR protocol (A) was performed according to Traub et al. (2004) [47] and the second protocol (B) with some modifications of protocol A [47] as follows: in 25 µl volumes with the final mix containing 12.5 pmol of each primer, 1X buffer Mix (EmeralAmp® GT PCR Master Mix; Takara Bio Inc., Shiga, Japan), H_2_O and 2 µl of DNA. Samples were heated at 96 °C for 10 min, 95 °C for 45 s, 59 °C for 40 s, 72 °C for 1 min for 10 cycles, then followed by 30 cycles of 95 °C for 45 s, 58 °C for 40 s, 72 °C for 60 s and 1 cycle of 72 °C for 7 min. In addition, the *U. stenocephala* adult DNA was used as positive control in all the PCR runs performed in the study.

The purified PCR products were sequenced in both forward and reverse directions and analyzed using Chromas 2.6.6 software. They were then compared with the NCBI/GenBank database using the Basic Local Alignment Search Tool (BLAST) and ClustalW software, for the discrimination between the two different hookworm species (*A. caninum* and *U. stenocephala*).

### Statistical analyses

Statistical analyses were considered only for the dogs that resulted positive to the hookworm infections; other co-infections were excluded. Hence, dogs were classified into five age groups: puppies (up to 12 months); young dogs (13–36 months); adult dogs (37–72 months); old dogs (73–120 months); and very old dogs (> 120 months). In addition, dogs were classified into three groups (small, medium and large) based on the breed size and into five groups (A, B, C, D, E) based hookworms egg excretion (A = 2–50 EPG; B = 52–100 EPG; C = 102–500 EPG; D = 502–1000 EPG; E ≥ 1002 EPG). Positivity for hookworms was analyzed in association with the above-mentioned variables (gender, age dog breed size and egg excretion) using univariate and logistic regression analysis. Data regarding the year of analysis were excluded from statistical analyses because the dog population of each year was variable. Moreover, data regarding previous antiparasitic treatments were very sparse and incomplete, therefore were also excluded from the statistical analyses.

Any association was considered significant at *P* < 0.005. The prevalence and the 95% confidence intervals (95%CI) were calculated using the free online software «Sample Size Calculator» (Creative Research Systems, CA, USA). All statistical analyses were performed using the SPSS® software (version 22,0, IBM Corporation, Armonk, USA).

## Data Availability

The datasets used and/or analyzed during the current study are available from the corresponding author on reasonable request.

## Abbreviations

ESCCAP: European Scientific Counsel Companion Animal Parasites
EPG: Eggs per gram of feaces
OPG: Oocysts per gram of feaces
LPG: Larvae per gram of feaces
CPG: Cysts per gram of feaces
